# *Vitis vinifera* L. Filled Chitosan Composite Microspheres for Adsorption of Toxic Methylene Blue Dye from Aqueous Solutions: Isotherm, Kinetic, Thermodynamic Studies and Error Analysis

**DOI:** 10.1021/acsomega.5c12678

**Published:** 2026-05-09

**Authors:** Ayse Erdogan Cakar, Birol Isik, Fatih Cakar

**Affiliations:** Department of Chemistry, Faculty of Arts & Sciences, 52999 Yildiz Technical University, Esenler, Istanbul 34220, Turkey

## Abstract

This study produced natural, sustainable, and efficient composite microbeads by incorporating *Vitis vinifera* L. (VF) biomass, regarded as a zero-waste product, and is transformed into chitosan (CS) biopolymer at various ratios, intended for use as an effective adsorbent material for the removal of dye (MB) from aquatic environments. The chitosan/biomass composite (CS-VF) was analyzed using FT-IR, SEM, EDS, XRD, and pH_pzc_ analyses. The dosage (0.01 to 0.30 g), pH (2 to 12), and duration (0 to 150 min) of CS-VF-30 composite microbeads were examined, and the optimal conditions for the adsorption process were established. The impact of salt content on the adsorption process was investigated using monovalent NaCl and divalent CaCl_2_ salts. The adsorption of MB by CS-VF-30 composite microbeads was accurately characterized by isotherms and adsorption kinetics, validating the Langmuir and *pseudo-second-order* kinetic models. The q_m_ value was ascertained to be 45.79 mg/g at 298 K by using the Langmuir isotherm model. This results from the electrostatic interaction, H-bonding, and π–π stacking interactions of the MB dye with the CS-VF-30 composite microbeads. Conversely, reusability experiments established that the composite microbeads can be utilized multiple times. This research demonstrates that CS-VF-30 composite microbeads serve as an effective bioadsorbent for the treatment of high-strength dye wastewater. Furthermore, the prepared composite microspheres were found to be highly effective in different water samples and could be reused repeatedly. The results demonstrate a sustainable and eco-friendly method for producing chitosan/biomass composite adsorbents for the extraction of cationic dyes from polluted water.

## Graphical abstract

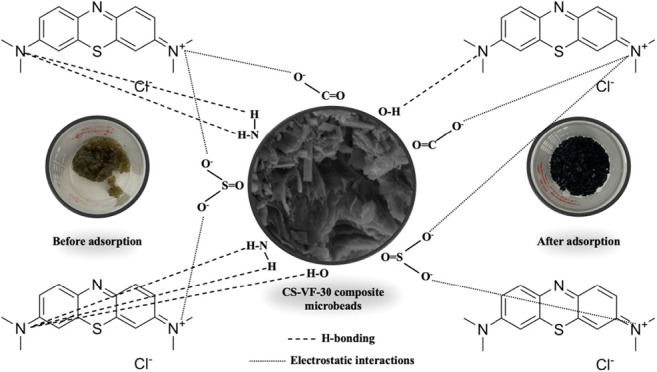

## 1 Introduction

The pollution of water resources by various pollutants is one of the most significant environmental issues. In leather, paper, textiles, and many other industries, dyes are commonly used to color products. About 700,000 tons of textile dyes, or 10,000 different types, are produced each year, according to the Textile Color Index.^1^ However, they can cause significant environmental and water resource problems. Furthermore, the delicate balance of the marine ecosystem is upset when dye supplies are released into the water without being treated, which lowers the water resources’ ability for photosynthetic activity. The discharge of dyes into the aquatic environment can have detrimental effects on the aquatic ecosystem, even at extremely low concentrations.^2–4^ Methylene blue (MB), one of these, is extremely resistant to environmental deterioration and has long-lasting effects. MB is suspected to having carcinogenic potential and may induce tissue damage, vomiting, shock, and elevated heart rate.^5,6^ Dyes must be removed from the aquatic ecosystem in a suitable and effective manner because of their severe adverse impacts.

Many methods have been applied to remove dyes from wastewater. Although energy is needed for biological and chemical processes like photocatalysis,^7^ oxidation–reduction,^8^ coagulation and flocculation,^9^ biodegradation,^10^ and electrochemistry,^11^ physical processes like adsorption,^12^ ion exchange,^13^ and membrane filtration^14^ are more practical and efficient. Because it is simple to use, inexpensive, sustainable, eco-friendly, and practical, researchers choose the adsorption approach over the others.^15–17^ However, alternative methods, such as photocatalytic degradation, release secondary pollutants like hydrocarbons, phenol, CO_2_, and other organic substances.^18,19^

In many nations, studies on biomass utilization have been carried out to combat the buildup of biomass waste, particularly agricultural waste. Through the creation of carbonaceous materials to extract different pollutants from wastewater, biomass utilization is extensively used for environmental sustainability in addition to energy production, such as the production of ethanol and methane.^20,21^ Activated carbon, zeolite, activated alumina, and bentonite clay are frequently used compounds for adsorption process.^22,23^ Among these components, activated carbon is most preferred, but it is limited by its high production cost and regeneration during the adsorption process.^24^ Recently, researchers have conducted many studies on the adsorption performance of sustainable and abundant lignocellulosic materials such as apple pomace,^25^ sugar pulp,^26^ sawdust,^27^ biopolymers,^28^ wheat straw,^29^ guava plants,^30^ St. John’s wort,^31^ etc.

Among lignocellulosic materials, chitosan (CS) is made up of the acetylated unit β-(1→4)-2-acetamido-d-glucose and the deacetylated unit β-(1→4)-2-amino-2-deoxy-d-glucose.^32,33^ This significant biopolymer’s abundance, nontoxicity, biocompatibility, and biodegradability have made it an affordable, efficient, and environmentally benign adsorbent. The applicability of CS as a promising biopolymer-based adsorbent for a variety of organic dyes is supported by the presence of −OH, −NH_2_, and −C=O in its structure. Better stability and superior adsorption performance are also obtained when chitosan is blended with a solid adsorbent, such as different biomasses.^34–36^

Vine leaves (*Vitis vinifera* L.), which have broad leaves 5–20 cm long, are a plant with woody branches that shed leaves in the winter and grow by wrapping around the plants around them. *Vitis vinifera* L., thought to originate from Southwest Asia, is rich in valuable compounds such as flavonoids, anthocyanins, procyanidins, tannins, enzymes, and vitamins. Grape leaves have been processed in various ways since ancient times. *Vitis vinifera* L., which is important in terms of industry, has a wide place in medicine due to its biological activities such as hepatoprotective, spasmolytic, hypoglycemic, and vasorelaxant effects, antibacterial, antifungal, anti-inflammatory, antinociceptive, antiviral, and especially antioxidant properties.^37,38^

The significance of this research lies in the fact that it is the very first time that chitosan-based composite microspheres (CS-VF) containing MB have been evaluated by using VF biomass as a functional filler. There have been several other agricultural wastes that have been included in the biopolymer matrix; however, the incorporation of VF powder into CS microspheres and the synergistic contribution that it makes to adsorption capacity have not been described before. The purpose of this research is to address the fundamental problem of generating low-cost, biodegradable, and highly efficient sorbents within a framework that emphasizes zero waste. This is accomplished by making use of an abundant viticulture byproduct that is frequently underused. The adsorption performance of the generated CS-VF composite microspheres against hazardous MB dye was carefully adjusted and modeled. This was done to disclose structure–function correlations. The structure–function relationships were revealed through comprehensive characterization using techniques such as FT-IR, SEM, EDS, pH_pzc_, and XRD. For presenting an innovative, ecologically friendly, and mechanistically supported solution for the treatment of dye-contaminated wastewater, extensive kinetic, isotherm, thermodynamic, and error analyses were carried out. These analyses were performed to precisely establish the paths of the adsorption.

## 2 Materials and Methods

### 2.1 Chemicals and Instrumentation

The chemicals and instrumentation used in this study are listed in Table 1.

**Table 1:** Chemicals and Instrumentation Used In This Work

chemicals	formula	source	molecular mass	purity
chitosan		Sigma-Aldrich	medium molecular weight	
methylene blue	C_16_H_18_ ClN_3_S	Sigma-Aldrich	319.85	85.0%
calcium chloride	CaCl_2_	Sigma-Aldrich	110.98	≥97.0%
acetic acid	CH_3_COOH	Sigma-Aldrich	60.05	≥99.7%
hydrochloric acid	HCl	Merck	36.46	37.0%
sodium hydroxide	NaOH	Sigma-Aldrich	39.997	

instrumentation devices	model	analysis purpose
FT-IR	Nicolet IS10 Thermo FT-IR	functional groups
SEM	Zeiss EVO LS10	morphology
EDS	Zeiss EVO LS10 EDAX	surface elemental analysis
pH meter	Ohaus starter 3100	pH optimizations
UV–vis	peak analysis C7200	removal %

### 2.2 Preparation of Composite Microspheres

The *Vitis vinifera* L. used in the experiment were supplied from the villages of Sarıgöl district of Manisa province, Türkiye. The stems of the vine leaves were separated from the leaves and washed under tap water. The *Vitis vinifera* L. were boiled in some water to remove impurities. The leaves were blanched to prevent any changes in the phenolic and other structural values of the *Vitis vinifera* L. The drained *Vitis vinifera* L. were dried under sunlight. After being ground into powder, it was passed through an 80-mesh sieve and dried in an oven at 70 °C.

To create composite microspheres, 2% by weight CS with a 75% deacetylation degree was gelatinized in 2% acetic acid. Following that, CS was mixed with 10, 20, and 30% by weight of *Vitis vinifera* L. biomass until it was uniform. To guarantee the development of microspheres, the homogeneous gel was then dropped into a 1 N NaOH solution from a predetermined height using a syringe. After that, the produced microspheres were stored for use in adsorption experiments, after being carefully cleaned with distilled water until their pH was neutral. Figure 1 illustrates the composite microspheres’ preparation process.

**Figure 1:**
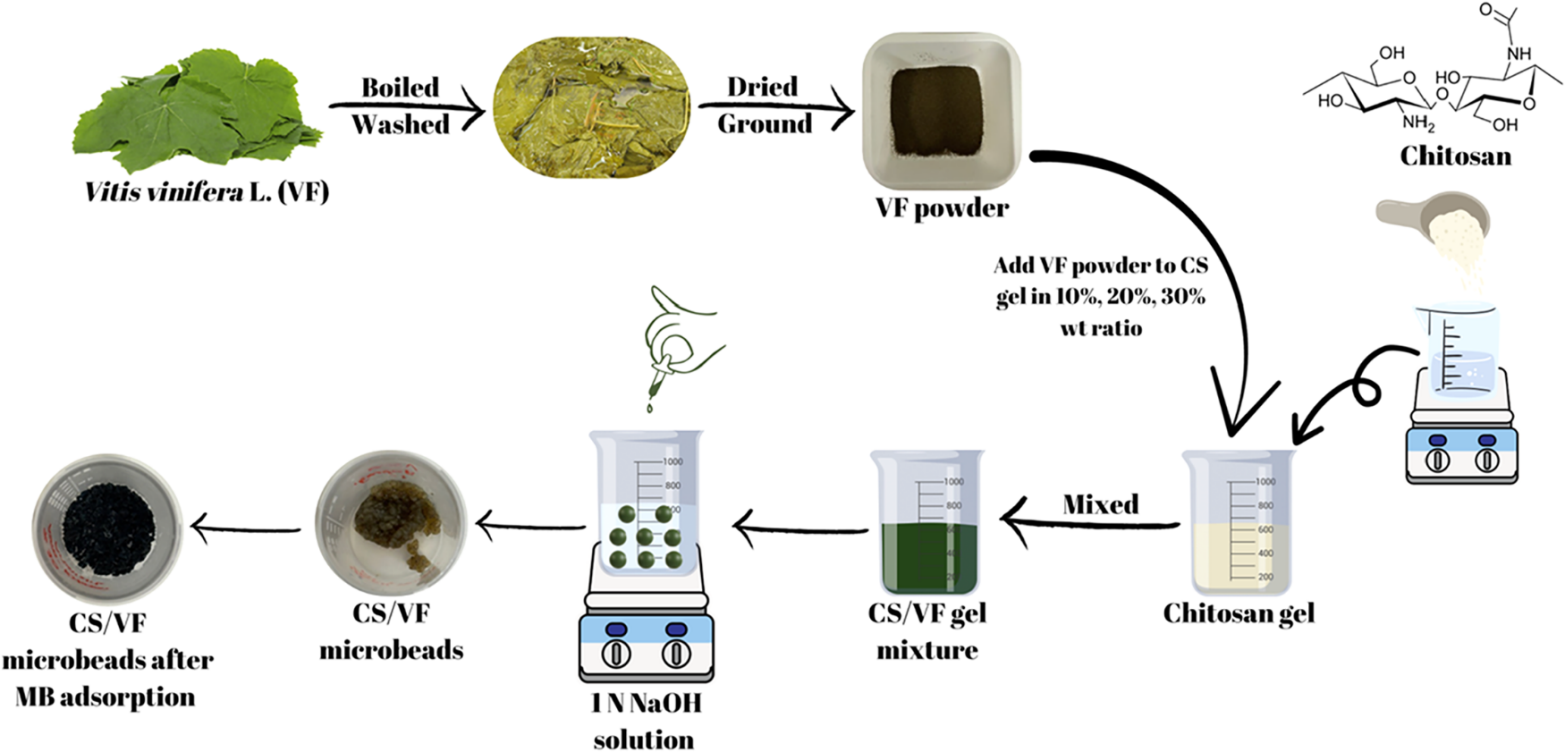
Preparation scheme of CS/VF composite microspheres as an adsorbent.

### 2.3 Batch Adsorption Tests

Batch adsorption studies were carried out in 100 mL conical bottles with a 50 mL MB dye solution volume and an initial MB dye concentration range of 10–50 mg/L with a 0.1 g/50 mL adsorbent dosage until equilibrium was reached. To identify the ideal parameters for the experiment, tests were conducted with adsorbent dosages of 0.01 and 0.30 g/50 mL, solution pH ranges of 2–12, initial MB dye concentrations ranging from 10 to 50 mg/L, contact times of 0–150 min, and stirring speeds of 120 rpm. Using solutions of 0.1 M HCl and 0.1 M NaOH, we brought the pH values of the solutions to the appropriate levels. Experimental studies were conducted in the concentration range of 10–50 mg/L, a contact period of 90 min, pH 7, and temperatures of 298, 308, and 318 K to perform the adsorption equilibrium studies and ascertain the isotherm parameters. Kinetic tests were performed at various temperatures with a concentration of 20 mg/L and a contact period ranging from 0 to 150 min to examine the adsorption mechanism. In batch adsorption experiments were performed in triplicate, and results were expressed as the mean ± standard deviation. Error bars in all graphs represent the standard deviation obtained from three independent measurements (*n* = 3). All experimental supernatant solutions were analyzed using a UV–vis spectrophotometer set to 663 nm. Equations 1, 2, and 3 were utilized to compute the % dye removal, the adsorption capacity at equilibrium, and the adsorption capacity after time “*t*”, based on the data that was acquired.^39–41^

1 $$\mathrm{Removal}\%=\frac{\left(C_{o}-C_{e}\right)}{C_{o}}\times 100$$

2 $$q_{e}=\left(C_{o}-C_{e}\right)\times \left(V/m\right)$$

3 $$q_{t}=\left(C_{o}-C_{t}\right)\times \left(V/m\right)$$

In these equations, *C*_o_, *C*_e_, and *C*_*t*_ are the initial, equilibrium, and at “*t*” time concentration (mg/L), respectively, *V* is the volume of the dye solution (0.05 L), and *m* is the adsorbent dosage (g).

To determine the isotherm parameters and assess the adsorption process, adsorbent performance, and adsorbent operation, the raw data was fitted to the Langmuir,^42^ Freundlich,^43^ Dubinin–Radushkevich (D-R),^44^ and Temkin^45^ isotherm models, and the outcomes were assessed. Table 2 displays the nonlinear equations for these isotherm models. Additionally, the raw data was subjected to four distinct kinetic models, *pseudo-first order*,^46^ *pseudo-second order*,^47^ intraparticle diffusion, and Boyd, to assess the adsorption mechanism and compute rate constants. Table 2 details these models’ nonlinear equations. Additionally, thermodynamic parameters were computed using the formulas shown in Table 2 utilizing information gathered from experiments carried out at various temperatures.^48,49^ To assess isotherm and kinetic models, experimental data were subjected to nonlinear regression and error analysis methodologies.

**Table 2:** Nonlinear Isotherm, Kinetic, and Thermodynamic Model Equations Used in This Study

isotherm models			
model	eq no	nonlinear equation	parameters
Langmuir	(4)	4 $q_{e}=\frac{q_{m}\times K_{L}\times C_{e}}{1+K_{L}\times C_{e}}$	*q*_e_: adsorption capacity in equilibrium (mg/g)
			*q*_m_: maximum adsorption capacity (mg/g)
			*K*_*L*_: Langmuir constant (L/mg)
Freundlich	(5)	5 $q_{e}=K_{F}\times C_{e}^{\left(1/n\right)}$	*K*_*F*_: Freundlich constant (L/mg)
			*n*: Freundlich isotherm constant
D-R	(6)	6 $$\begin{array}{l} q_{e}=q_{D-R}\times e^{-\beta \times ({R\times T\times \ln\left(1+\frac{1}{C_{e}}\right))}^{2}}\\ E_{D-R}={\left(2\beta \right)}^{-0.5} \end{array}$$	*q*_D‑R_: theoretical adsorption capacity (mg/g)
			*R*: ideal gas constant (8.314 J/mol.K)
			*E*_D‑R_: adsorption energy (kJ/mol)
Temkin	(7)	7 $q_{e}=\frac{R\times T}{b_{T}}\times \ln\left(K_{T}\times C_{e}\right)$	*b_T_*: fluctuation of adsorption energy (J/mol)
			*K_T_*: binding equilibrium value (L/mg)
kinetic models			
*pseudo-first order*	(8)	8 $q_{t}=q_{e}\times \left(1-e^{-k_{1}\times t}\right)$	*k*_1_: first-order rate constant (min^–1^)
*pseudo-second order*	(9)	9 $q_{t}=\frac{q_{e}^{2}\times k_{2}\times t}{1+q_{e}\times k_{2}\times t}$	*k*_2_: second-order rate constant (g/mg min)
*intraparticle diffusion*	(10)	10 $q_{t}=k_{d}t^{0.5}+C$	*k*_d_: intraparticle diffusion rate constant (mg/g min^0.5^)
			*C*: the constant linked with the thickness of the boundary layer (mg/g)
*Boyd*	(11)	11 $B_{t}=-0.4977-\ln\left(1-q_{t}/q_{e}\right)$	*B*_*t*_: Boyd constant linked with the fraction of MB
thermodynamics			
12 $K_{e}^{o}=\frac{1000\times K_{L}\times M_{\mathrm{MB}}\times {\left[\mathrm{Adsorbate}\right]}^{o}}{\gamma }$			*K*_e_^o^: thermodynamic equilibrium constant
			*M*_MB_: molecular mass of MB dye (g/mol)
			γ: dimensionless separation factor
			[*Adsorbate*]^o^: standard adsorbate concentration
13 $\ln K_{e}^{o}=-\frac{\Delta G^{o}}{R\times T}=\frac{\Delta S^{o}}{R}-\frac{\Delta H^{o}}{R}\times \frac{1}{T}$			*K*_e_^o^: thermodynamic equilibrium constant
14 $\Delta G^{o}=\Delta H^{o}-T\times \Delta S^{o}$			Δ*H* ^o^: enthalpy (kJ/mol)
			Δ*S* ^o^: entropy (J/mol/K)
			Δ*G* ^o^: Gibbs free energy (kJ/mol)
15 $\ln k_{2}=\ln A-\frac{E_{a}}{R\times T}$			*E*_a_: activation energy (kJ/mol)
			*A*: Arrhenius constant
error functions			
16 $\mathrm{SSE}=\sum_{i=1}^{n}{\left(q_{e,\exp}-q_{e,\mathrm{cal}}\right)}^{2}$			*q*_e,cal_: estimated adsorption capacity (mg/g)
17 $\mathrm{ARE}=\frac{100}{n}\sum_{i=1}^{n}\left|\frac{q_{e,\mathrm{cal}}-q_{e,\exp}}{q_{e,\exp}}\right|$			
18 $\mathrm{SD}=\sqrt{\frac{1}{n}\sum_{i=1}^{n}{\left(q_{e,\mathrm{cal}}-q_{e,\exp}\right)}^{2}}$			*q*_e,exp_: experimental adsorption capacity (mg/g)
19 $\chi^{2}=\sum_{i=1}^{n}\frac{{\left(q_{e,\exp}-q_{e,\mathrm{cal}}\right)}^{2}}{q_{e,\mathrm{cal}}}$			
21 $\mathrm{SD}=\sqrt{\frac{1}{n}\sum_{i=1}^{n}{\left(q_{e,\mathrm{cal}}-q_{e,\exp}\right)}^{2}}$			

The nonlinear least-squares method was employed to fit the data obtained to the models, and Microsoft Excel software was employed to calibrate the data. Statistical metrics, including SSE (sum of squared errors), ARE (absolute relative error), SD (standard deviation), and χ^2^ (chi-square), were implemented to evaluate the model fit for quality. The primary criteria for assessing the veracity of the model were statistical measurements. The mathematical procedures employed and the statistical parameters that were computed are elaborated in Table 2.^50,51^

## 3 Results and Discussion

### 3.1 Characterization of CS-VF-30 Composite Microbeads

The CS-VF-30 composite microbeads’ surface functional groups were examined using FT-IR investigations conducted in the 900–4000 cm^–1^ wavenumber range. Figure 2 displays the CS-VF-30 composite microbeads’ spectra both prior to and following the adsorption process. −OH stretching, C=O stretching, N–H stretching, −CH_3_ symmetric deformations, S=O stretching, and C–O stretching were the causes of the peaks that were seen at 3285, 1732, 1634, 1393, 1131, and 1023 cm^–1^, respectively. The peaks were found to have undergone a significant shift and to have lost some of their strength following the sorption process.^52,53^ The identical peaks were found at 3289, 1645, 1558, 1375, 1150, and 1027 cm^–1^, respectively, following the adsorption procedure. Electrostatic interactions between the MB dye molecules and the surface of the composite microbeads are responsible for the shifts in the peaks of the carboxyl, amine, sulfate, and hydroxyl functional groups. This could also suggest that during the adsorption process, these functional groups serve as adsorption sites.^54,55^ While other peaks transitioned to higher wavelengths, the band at 1393 cm^–1^ migrated to a lower wavelength of 1375 cm^–1^ subsequent to the adsorption process. This alteration can be ascribed to the torsional characteristics of this vibrational mode, exhibiting heightened electron density and enhanced hydrogen bonding surrounding the −CH_3_/–COO^–^ deformation medium upon complexation with MB. Such interactions alleviate torsional vibrations, diminish the force constant, and so move the band to a lower frequency.^56–58^

**Figure 2:**
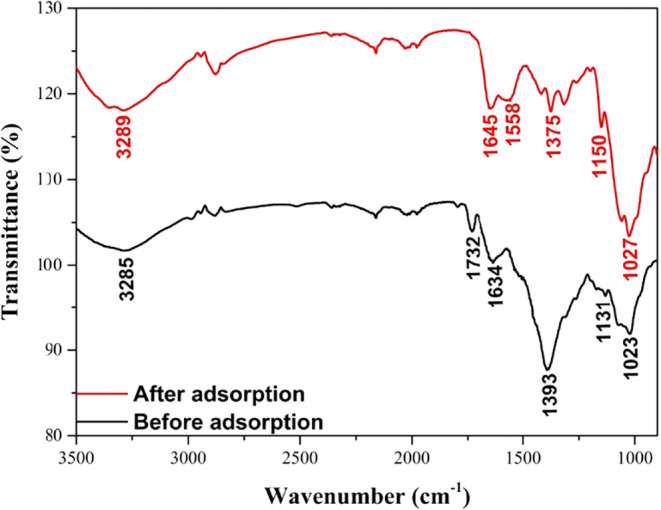
FT-IR spectra of CS-VF-30 composite microbeads before and after MB dye removal.

Figure 3 presents SEM images of CS-VF-30 composite microbeads before and after dye removal. Before dye removal, the CS-VF-30 composite microbeads exhibited a complex, slitlike, textured, and porous surface favorable for the retention of MB dye. Additionally, preadsorption SEM analysis also revealed a fibrous structure due to the addition of VF within the CS. This surface is optimized for effective adsorption. On the other hand, EDS analysis results showed that the structure consists of C, N, O, and S, as also seen in the FT-IR spectrum. After extraction of the MB dye from wastewater, the surface of the CS-VF-30 composite microbeads exhibited improved smoothness and homogeneity with significant filling of voids and concealment of surface defects by the dye. EDS mapping analysis after adsorption revealed the presence of the Cl element from the MB dye in the structure, indicating successful adsorption.

**Figure 3:**
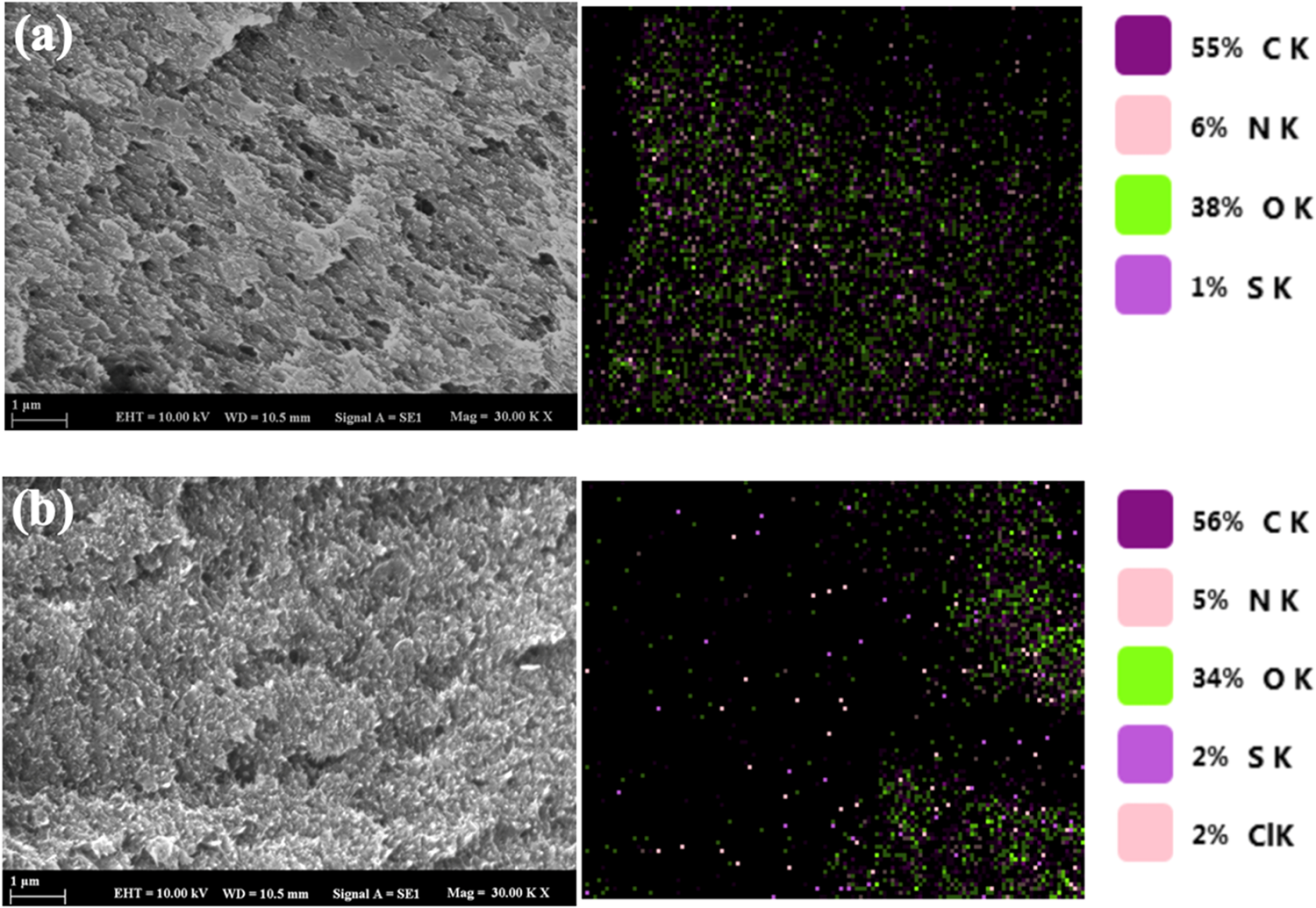
SEM images and EDS mapping figures of CS-VF-30 composite microbeads before (a) and after (b) removal of MB dye.

The zero charge point (pH_pzc_) of CS-VF-30 composite microbeads was determined by experiments using the salt addition method. According to the salt addition method, also used by Teweldebrihan et al., 0.2 g of CS-VF-30 composite microbeads was added to 50 mL (0.1 M) of sodium nitrate salt solution with an initial pH between 2 and 12. The pH values of the solution were adjusted using 0.1 M HCl and 0.1 M NaOH solutions and an Ohaus starter 3100 pH meter.^59^ To determine the final pH (pH_final_), the solutions adjusted for various pH levels were kept on a shaker at 298 K for 24 h. CS-VF-30 composite microbeads’ pH_pzc_ value was determined by plotting the original pH values versus ΔpH (pH_final_ – pH_initial_). Determining the pH at which the surface exhibits electrostatic charge neutrality requires knowing the CS-VF-30 composite microbeads’ pH_pzc_ value. As shown in Figure 4, the CS-VF-30 composite microbeads’ pH_pzc_ value was determined to be 7.08. It was found that the CS-VF-30 composite microbeads had a roughly neutral surface. The CS-VF-30 composite microbeads’ surface has a positive charge below pH_pzc_, which makes it easier for anionic dyes to adsorb. On the other hand, it has a negative charge above the pH_pzc_, which facilitates the adsorption of cationic dyes.

**Figure 4:**
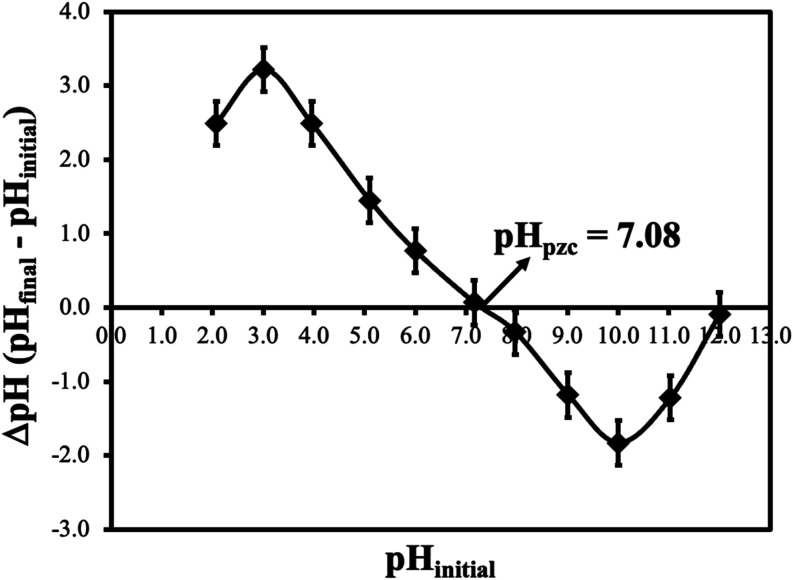
PH_pzc_ graph of CS-VF-30 composite microbeads. Data were presented as mean ± standard deviation (*n* = 3).

### 3.2 Effect of Treatment Parameters

Various quantities of CS-VF-30 composite microbeads (ranging from 0.01 to 0.30 g) were employed for the adsorption of 50 mL of a 20 mg/L MB dye solution at ambient temperature and pH 7 for a duration of 150 min (Figure 5a). The initial and ultimate absorbance of the MB dye were measured using a UV–vis spectrophotometer after 60 min. It was noted that 0.1 g of CS-VF-30 composite microbeads exhibited the highest adsorption owing to an increased number of active sites on the adsorbent. The limited number of active sites at lower microbead quantities resulted in a reduced adsorption percentage, whereas the adsorption performance improved with an increase in quantity. The adsorption efficiency improved from 53.03 to 93.45% when the quantity of microbeads increased from 0.01 to 0.30 g. The adsorption capacity diminished from 53.03 to 3.12 mg/g. The reduction in adsorption capacity may result from the aggregation of active moieties on the composite microbeads’ surface, hence diminishing adsorption effectiveness.^60–62^

**Figure 5:**
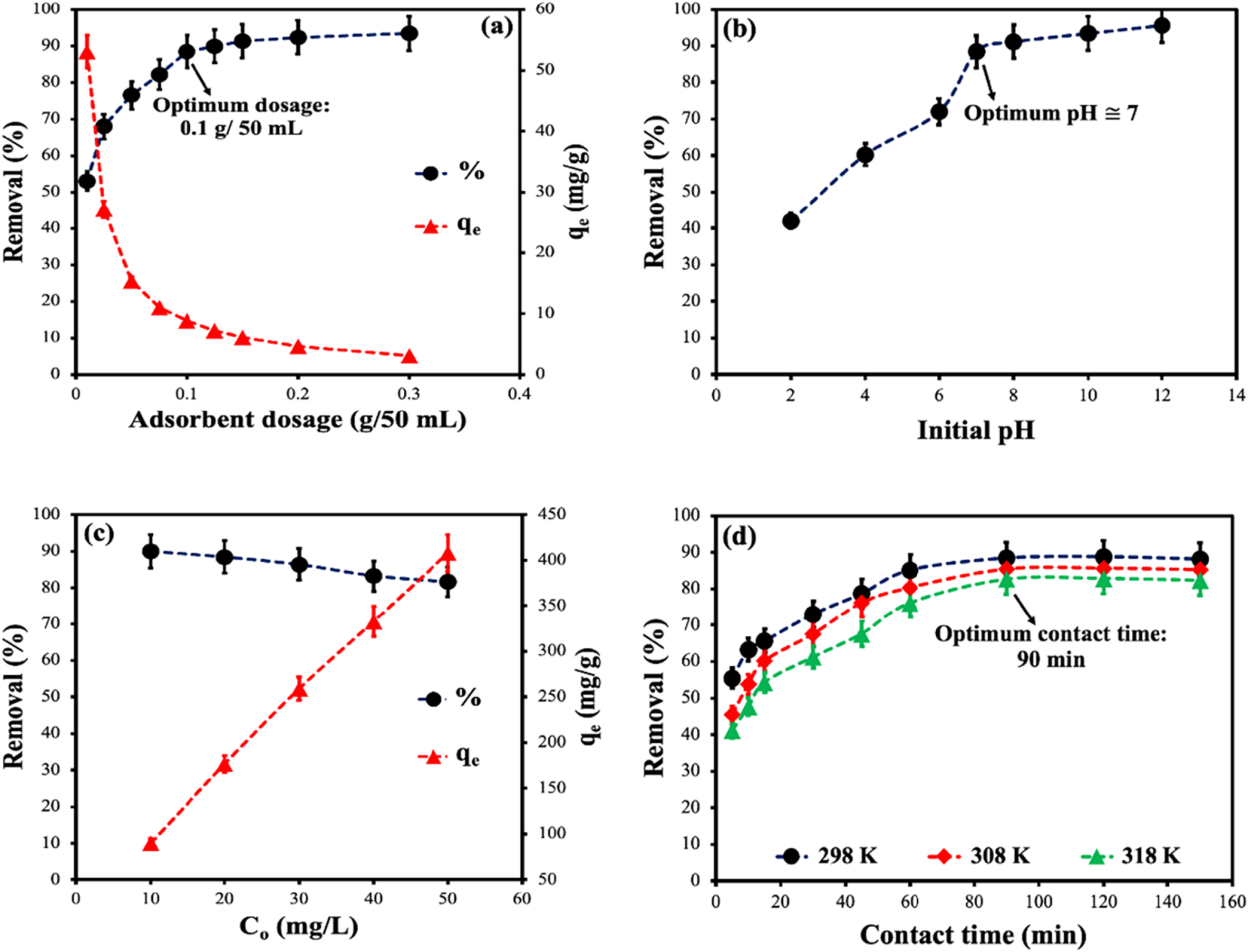
Effect of treatment parameters on MB dye adsorption using CS-VF-30 composite microbeads: (a) adsorbent dosage, (b) initial concentration, (c) initial pH, and (d) contact time. Data were presented as mean ± standard deviation (*n* = 3).

The pH of the adsorbent is a crucial factor in the adsorption process as it alters the surface charge of the adsorbent and the chemical structure of the dyes.^63,64^ The influence of pH (Figure 5b) on the adsorption capacity of CS-VF-30 composite microbeads was examined by utilizing 0.1 g of adsorbent and 50 mL of a 20 mg/L MB solution for a duration of 60 min. All experiments were conducted at pH levels of 2, 4, 6, 8, 10, and 12 at ambient temperature. The pH was modified utilizing a dilute HCl or NaOH solution. The outcomes are depicted in Figure 5b. The quantity of adsorbed MB was significantly influenced by the initial pH of the solution. The highest MB adsorption occurred at pH 7 with all three adsorbents; however, the adsorption % was minimal under acidic conditions, peaked under neutral conditions, and gradually increased with rising pH levels. Adsorption was maximized in a basic environment relative to that in an acidic environment. This occurs due to the generation of a greater negative surface charge on the adsorbent, which enhances the adsorption of positive MB dyes. At elevated pH levels (pH > pH_pzc_ = 7.08), the repulsive contact between the hydroxyl group and the negatively charged surface diminishes adsorption efficacy. Conversely, at reduced pH levels (pH < pH_pzc_ = 7.08), the surface charge of the adsorbent becomes increasingly positive, hindering the adsorption of positively charged MB dyes, hence diminishing adsorption efficiency.

Various concentrations of MB dye (10, 20, 30, 40, and 50 mg/L) were examined at pH 7 and ambient temperature for a duration of 60 min. Figure 5c clearly illustrates that the adsorption rate diminished as the concentration of the dye solution increased, which can be attributed to the saturation effect. At elevated concentrations of the dye solution, the adsorption sites on the composite microbeads may have reached saturation.^65,66^ At lower concentrations, the polymer surface exhibited a greater number of unoccupied adsorption sites, leading to an increased adsorption rate. The adsorption rate diminished, and the quantity of unoccupied sites declined as the concentration escalated. The adsorption percentage diminished from 89.98 to 81.56% when the concentration escalated from 10 to 50 mg/L, but the adsorption capacity augmented from 4.50 to 20.39 mg/g.

To assess the influence of time and equilibrium duration, 0.1 g of each adsorbent was introduced into a conical flask holding 50 mL of 20 mg/L MB dye, and the adsorption procedure was conducted. 2 mL aliquots of the reaction mixture were extracted at intervals from 0 to 150 min, and the variation in absorbance was measured by using a UV–visible spectrophotometer. The findings indicated that adsorption intensified over time, achieving equilibrium after 60 min. The initial swift rise in the quantity of adsorbed dye may be attributed to the initially more available unoccupied active sites, which subsequently reached saturation over time (Figure 5d). The CS-VF-30 composite microbeads had the highest adsorption percentage, at 87%. The adsorption performance diminished with a rising temperature, suggesting that the process was exothermic. Similar results were also found in studies in the literature.^67,68^

### 3.3 Effect of VF Additive

Figure 6 illustrates the impact that the incorporation of VF biomass into the structure of composite microspheres, expressed as a percentage of the total weight, has on the adsorption effectiveness of the mobile phase dye. Following an investigation of the constituents of the composite microspheres, it was found that the adsorption capabilities of CS and VF were, respectively, 24.75 and 26.36 mg/g. After investigating the constituents of the composite microspheres, it was discovered that they could remove MB dye in an efficient manner, and their capability was amazing. On the other hand, the adsorption capacity of the composite microspheres dramatically enhanced when the VF ratio in those microspheres increased. CS-VF-30 composite microspheres had an adsorption capacity of 45.79 mg/g when heated to 298 °C. This is 1.74 times greater than the adsorption capacity of the VF component, and it is 1.85 times higher than the adsorption capacity of the CS component compared to the adsorption capacity of the CS component. There was a considerable increase in the number of active elements that were necessary for adsorption because of the incorporation of VF into the CS template. The findings indicate that the newly synthesized CS/VF composites, which are both natural and sustainable, perform as extremely efficient adsorbents for the elimination of cationic contaminants from aqueous solutions. This is demonstrated by the fact that the composites are completely natural.

**Figure 6:**
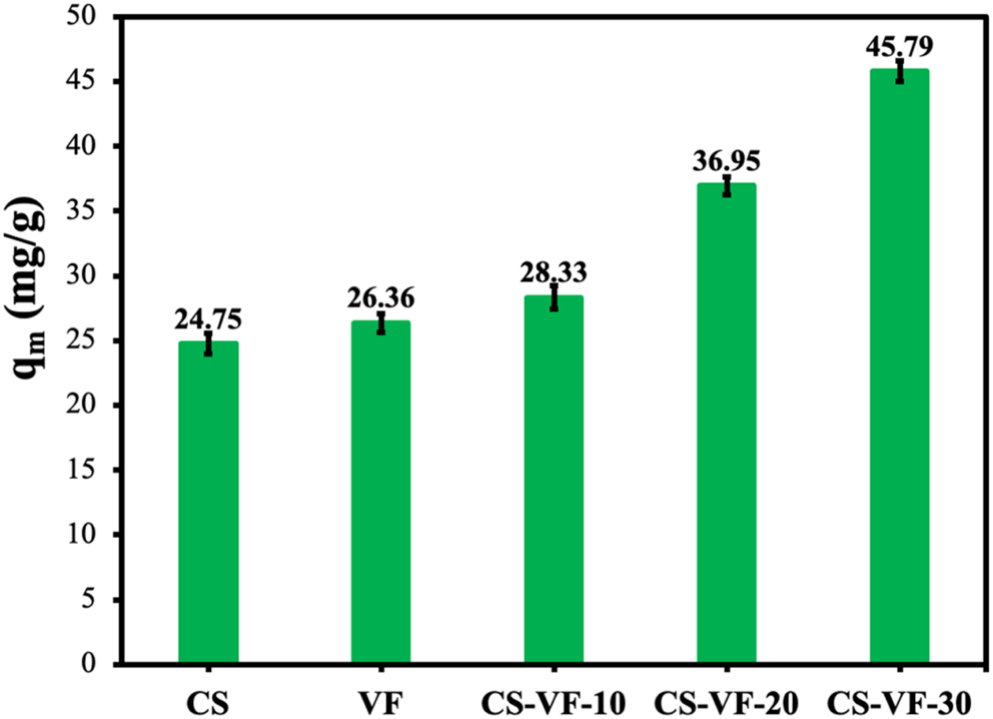
Effect of VF additive on MB dye adsorption at 298 K. Values represent the mean ± standard deviation of three independent experiments (*n* = 3).

### 3.4 Effect of Salination

Figure 7 illustrates how the MB adsorption process utilizing CS-VF-30 composite microbeads is affected by monovalent NaCl and divalent CaCl_2_ salts. When these salts were added to the solution, the adsorption efficiency significantly dropped. The percentage of MB eliminated dropped from 85.12 to 72.11% when NaCl was added and from 85.12 to 56.54% when CaCl_2_ was added. In addition to the reduction in electrostatic attraction between the positively charged MB molecules and the negatively charged regions on the surface of the CS-VF-30 composite microbeads, this decrease can be explained by the partial blockage of the active binding sites on the adsorbent surface caused by the presence of Na^+^ and Ca^2+^ ions. According to the findings, the adsorption mechanism heavily relies on electrostatic interactions.^69,70^

**Figure 7:**
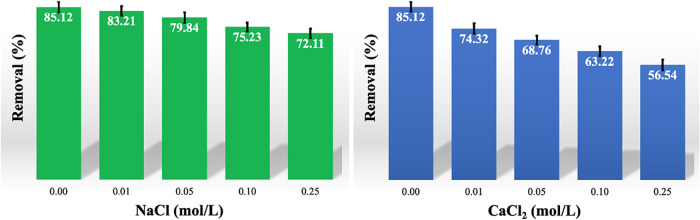
Effect of salination on the MB adsorption performance of CS-VF-30 composite microbeads. Error bars indicate standard deviation from triplicate measurements (*n* = 3).

### 3.5 Adsorption Equilibrium Tests

Adsorption isotherm models are a great method for characterizing the adsorption process and examining the underlying mechanisms.^71,72^ Isotherm curves depicting the adsorption of MB dye onto CS-VF-30 composite microbeads were illustrated at various temperatures, as presented in Figure 8a. Figure 8a illustrates that the isotherm curves conform to the L1 type isotherm model as per Giles’ isotherm classification^73^ across all temperature ranges. The equilibrium data for CS-VF-30 composite microbeads were analyzed using nonlinear Langmuir, Freundlich, D-R, and Temkin isotherm models to assess the adsorption characteristics.

**Figure 8:**
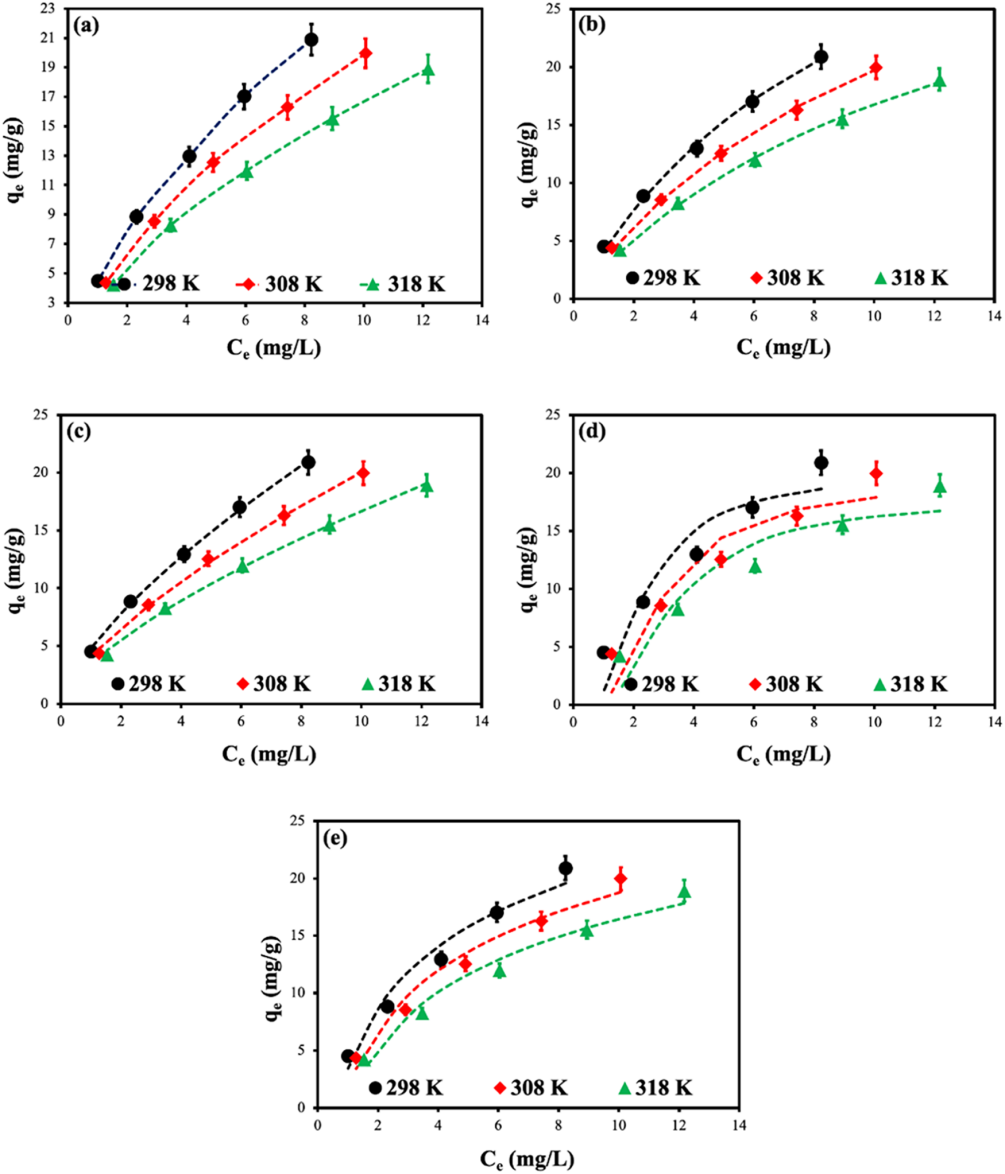
Isotherm curves (a) and nonlinear Langmuir (b), Freundlich (c), D-R (d), and Temkin (e) graphs of MB dye adsorption onto CS-VF-30 composite microbeads. Error bars indicate standard deviation from triplicate measurements (*n* = 3).

Figure 8 displays the nonlinear isotherm curves of the isotherm models shown in Microsoft Excel. The isotherm parameters were derived from the isotherm plots, and the findings are presented in Table 3. Table 3 indicates that, based on the correlation coefficient and the minimal error function values presented in Table 4, the Langmuir isotherm model is identified as the best appropriate model for MB adsorption onto CS-VF-30 composite microbeads. The maximal adsorption capacity (*q*_m_) derived from the Langmuir isotherm model was determined to be 45.79 mg/g at 298 K for the CS-VF-30 composite microbeads. Conversely, it was noted that the adsorption capacity was markedly enhanced with the augmentation of VF in the CS structure. The *q*_m_ value for VF biomass was 26.36 mg/g at 298 K, which rose nearly 1.74-fold to 45.79 mg/g in CS-VF-30 composite microbeads. This demonstrated that an increase in the quantity of VF within the structure corresponded to a substantial rise in the active moieties on the surface, hence markedly enhancing the adsorption performance. The Langmuir isotherm constant *K*_*L*_ ranged from 0.0713 to 0.1628 L/mg for all adsorbents, indicating a favorable adsorption process.

**Table 3:** Nonlinear Isotherm Parameters of MB Dye Adsorption onto CS, VF, CS-VF-10, CS-VF-20, and CS-VF-30 Composite Microbeads at Different Temperatures

	nonlinear Langmuir			nonlinear Freundlich			nonlinear D-R			nonlinear Temkin		
temperature	*q*_m_ (mg g^–1^)	*K*_*L*_ (L mg^–1^)	*R* ^2^	*K*_*F*_ (L mg^–1^)	*n*	*R* ^2^	*q*_*D‑R*_) (mg g^–1^)	*E*_*D*‑*R*_) (kJ mol^–1^)	*R* ^2^	*K*_*T*_ (L mg^–1^)	*b*_*T*_ (J mol^–1^)	*R* ^2^
298 K												
CS	24.75	0.1022	0.9974	3.406	1.847	0.9992	15.33	0.452	0.9951	0.8504	423.1	1.0000
VF	26.36	0.7490	0.9969	10.72	2.38	0.9987	19.99	1.87	0.9965	8.061	442.7	0.9999
CS-VF-10	28.33	0.0847	0.9982	3.161	1.68	0.9990	15.74	0.46	0.9934	0.8557	404.9	1.0000
CS-VF-20	36.95	0.1628	0.9991	5.891	1.61	0.9983	20.01	0.85	0.9916	1.935	336.5	1.0000
CS-VF-30	45.79	0.1003	0.9941	4.817	1.43	0.9983	20.14	0.72	0.9891	1.566	323.7	1.0000
308 K												
CS	21.45	0.0987	0.9981	3.048	1.924	0.9993	13.49	0.462	0.9970	0.8586	513.1	1.0000
VF	25.98	0.3773	0.9952	7.585	2.12	0.9997	17.97	1.41	0.9959	4.691	483.7	1.0000
CS-VF-10	26.92	0.0783	0.9973	2.877	1.68	0.9990	15.02	0.44	0.9938	0.7764	436.1	1.0000
CS-VF-20	33.68	0.1388	0.9991	5.014	1.67	0.9987	18.90	0.72	0.9903	1.533	367.1	1.0000
CS-VF-30	43.25	0.0837	0.9959	4.018	1.43	0.9982	19.31	0.62	0.9879	1.241	344.1	1.0000
318 K												
CS	18.13	0.0935	0.9972	2.598	1.985	0.9993	11.62	0.456	0.9985	0.8308	632.7	1.0000
VF	23.72	0.2367	0.9958	5.568	2.11	0.9995	15.94	1.07	0.9971	2.781	536.5	1.0000
CS-VF-10	23.02	0.0713	0.9989	2.429	1.74	0.9991	13.06	0.41	0.9957	0.7028	523.9	1.0000
CS-VF-20	30.60	0.1108	0.9999	3.991	1.67	0.9987	17.12	0.63	0.9926	1.216	414.7	1.0000
CS-VF-30	39.34	0.0745	0.9958	3.451	1.46	0.9982	17.88	0.57	0.9907	1.061	379.2	1.0000

**Table 4:** Error Functions for the Adsorption of MB Dye onto CS, VF, CS-VF-10, CS-VF-20, and CS-VF-30 Composite Microbeads at Different Temperatures

	nonlinear Langmuir				nonlinear Freundlich				Nonlinear D-R				Nonlinear Temkin			
temperature	SSE	ARE	SD	*χ* ^2^	SSE	ARE	SD	*χ* ^2^	SSE	ARE	SD	*χ* ^2^	SSE	ARE	SD	*χ* ^2^
298 K																
CS	2.641	8.204	0.7267	0.2864	7.246	13.81	1.204	0.7952	6.501	15.87	1.141	1.792	1.974	4.257	0.6284	0.1544
VF	0.8022	3.379	0.4006	0.0637	3.789	8.903	0.8705	0.4354	16.63	16.71	1.824	2.604	0.3761	2.717	0.2743	0.0416
CS-VF-10	0.8873	3.669	0.4212	0.0792	3.361	8.683	0.8197	0.3601	10.82	19.43	1.471	3.589	1.229	5.464	0.4957	0.1442
CS-VF-20	0.0142	0.4009	0.0533	0.0012	1.502	5.534	0.5479	0.1721	17.79	20.04	1.887	5.992	2.486	7.292	0.7052	0.3113
CS-VF-30	0.3293	2.747	0.2566	0.0419	0.2225	2.314	0.2109	0.0301	21.55	22.08	2.076	10.11	5.501	10.28	1.049	0.6272
308 K																
CS	0.9277	4.389	0.4307	0.0997	3.829	10.19	0.8751	0.4573	6.726	16.16	1.159	1.448	0.8509	2.884	0.4125	0.0711
VF	4.037	8.629	0.8986	0.4373	0.6563	3.719	0.3623	0.0851	27.74	22.41	2.356	5.394	4.153	7.663	0.9114	0.3553
CS-VF-10	0.6132	3.916	0.3502	0.0657	2.845	8.807	0.7543	0.3351	9.211	18.58	1.357	3.016	0.7622	4.491	0.3904	0.1002
CS-VF-20	0.3155	1.645	0.2512	0.0211	2.761	7.172	0.7429	0.2748	16.37	20.28	1.809	6.649	1.933	6.952	0.6218	0.2513
CS-VF-30	0.1575	1.627	0.1775	0.0161	0.3178	2.737	0.2521	0.0456	19.08	22.11	1.953	10.45	4.182	9.634	0.9146	0.5114
318 K																
CS	0.0646	1.635	0.1137	0.0111	1.409	7.712	0.5309	0.2159	5.538	14.77	1.052	0.9856	0.0655	1.209	0.1144	0.0073
VF	2.479	7.180	0.7041	0.2899	0.4485	3.356	0.2995	0.0661	23.72	22.09	2.178	4.011	2.627	6.661	0.7248	0.2463
CS-VF-10	0.3963	2.781	0.2815	0.0381	1.943	7.633	0.6235	0.2376	8.502	19.09	1.304	2.385	0.7314	4.732	0.3825	0.1033
CS-VF-20	0.1306	0.9161	0.1616	0.0089	1.268	5.451	0.5037	0.1701	15.03	20.48	1.734	5.078	1.311	5.768	0.5119	0.1721
CS-VF-30	0.2062	2.228	0.2031	0.0228	0.2556	2.896	0.2261	0.0425	18.25	22.05	1.910	7.805	3.469	8.849	0.8329	0.4168

The adsorption capacity of CS-VF-30 composite microbeads was assessed alongside other registered adsorbents to evaluate their competitiveness as bioadsorbents for MB sorption (refer to Table 5). The evaluated composite microbeads demonstrated considerable ability to absorb MB. This outcome concerning the removal of the dye substantiates that composite microbeads are a viable alternative to existing established adsorbents. The results underscore the efficacy of composite microbeads as a potent adsorbent for the removal of cationic dyes, including MB. This study indicates that composite microbeads can function as a legitimate alternative to several traditional adsorbents employed in color removal techniques. Composite microbeads serve as an economical and plentiful resource, augmenting environmental sustainability and economic feasibility. The application of composite microbeads in dye wastewater treatment exemplifies environmental sustainability and economic feasibility.

**Table 5:** Comparison of MB Dye Adsorption Capacities of VF, CS-VF-10, CS-VF-20, and CS-VF-30 Composite Microbeads with Other Composites Reported in the Literature

adsorbent	*q*_**m**_ (mg/g)	refs
CS-VF-30	45.79	this work
SRC/Cs	97.08	^74^
Chit/AILP-Kao	99.01	^75^
GO/CS	7.53	^76^
spent coffee/chitosan	75.76	^77^
CH_1_KBC	45.60	^78^
CuMn_2_O_4_/chitosan	54.05	^79^
Fe-TMC-N	42.00	^80^
Cs20B80	55.27	^81^
GO@CS	23.26	^82^

Table 3 reveals that the ″*n*″ value in the Freundlich isotherm plot ranged from 1.43 to 2.18 for all adsorbents, signifying good adsorption of MB onto the adsorbents. Upon analyzing the correlation coefficient and the error functions presented in Table 4, it can be inferred that the Langmuir model is more suitable for the adsorption process than the Freundlich model. The average D-R free energy was determined to vary from *E*_D‑R_ = 0.41 kJ/mol to E_D‑R_ = 1.87 kJ/mol, substantiating that the adsorption of MB dye onto the adsorbents transpires physically in accordance with the D-R isotherm model.^83^ The Temkin curve illustrated in Figure 8d indicates that the model posits that the heat of adsorption is variable. The reduction occurs because of the interaction between the adsorbent and the adsorbate during the adsorption process. The subsequent values were assessed. The equilibrium binding constant for CS-VF-30 composite microbeads is 1.566 L/mg at 298 K, with a Temkin constant *b*_*T*_ of 323.7 J/mol, as determined by the maximal binding energy (*K*_*T*_). A number beyond zero signifies the heat liberated during the adsorption process, denoting an exothermic adsorption phenomenon.^84^ Overall, the Langmuir isotherm model demonstrates superior fitting compared with the other three isotherm models, as evidenced by both the correlation coefficient and the error functions presented in Table 4.

### 3.6 Adsorption Kinetic Tests

The adsorption process mechanisms are commonly described using *pseudo-first-order*, *pseudo-second-order*, intraparticle diffusion, and Boyd models, which were utilized to establish the adsorption kinetic parameters.^85–87^ The findings are shown in Figure 9. The models’ graphs were drawn using the equations in Table 2. These nonlinear graphs supplied the kinetic parameters. Table 6 presents a summary of the findings. Clear information about the adsorption process and any rate-limiting phases can be predicted by using this model. In general, the second-order kinetic model is a better model for MB dye adsorption since it assumes that a key element influencing adsorption rates is the rate of chemical interaction between MB dye molecules and CS-VF-30 composite microbeads. The experimental findings suited the *pseudo-second-order* kinetic model for CS-VF-30 composite microbeads well since the correlation coefficient values were nearly equal to unity.

**Figure 9:**
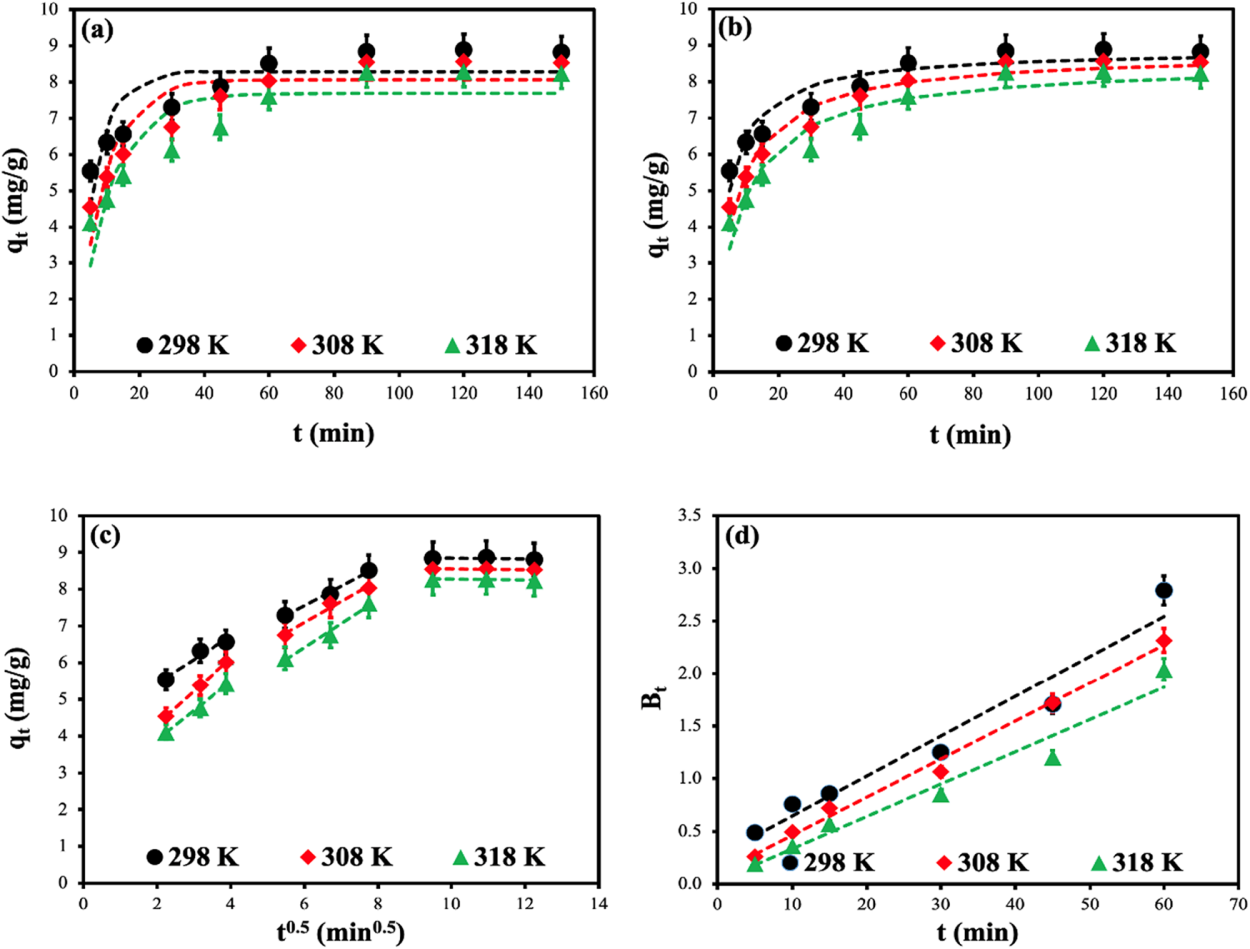
Nonlinear *pseudo-first-order* (a), *pseudo-second-order* (b), intraparticle diffusion (c), and Boyd (d) kinetic curves of MB dye adsorption onto CS-VF-30 composite microbeads. Error bars indicate standard deviation from triplicate measurements (*n* = 3).

**Table 6:** Kinetic Parameters for Adsorption of MB Dye onto CS, VF, CS-VF-10, CS-VF-20, and CS-VF-30 Composite Microbeads at Different Temperatures

	nonlinear *pseudo-first order*				nonlinear *pseudo-second order*				intraparticle diffusion								
temperature	*k*_1_ (min^–1^)	*q*_e,exp_ (mg/g)	*q*_e,cal_ (mg/g)	*R* ^2^	*k*_2_ (g/mg min)	*q*_e,exp_ (mg/g)	*q*_e,cal_ (mg/g)	*R* ^2^	*k*_d1_ (min^0.5^)	*C*_1_ (mg/g)	*R* ^2^	*k*_d2_ (min^0.5^)	*C*_2_ (mg/g)	*R* ^2^	*k*_d3_ (min^0.5^)	*C*_3_ (mg/g)	*R* ^2^
298 K																	
CS	0.1863	7.635	7.335	0.9998	0.0425	7.635	7.762	0.9999	0.7119	3.514	0.9734	1.022	0.8991	0.9999	1.022	0.3216	0.9842
VF	0.2421	9.635	9.264	0.9999	0.0484	9.635	9.709	0.9999	0.7144	5.510	0.9540	0.2701	7.399	0.9634	–0.0047	9.687	0.1626
CS-VF-10	0.2767	7.712	7.258	0.9999	0.0692	7.712	7.612	0.9999	0.3730	5.029	0.9912	0.3032	5.101	0.9991	–0.0195	7.913	0.4687
CS-VF-20	0.2871	9.016	8.576	0.9999	0.0644	9.016	8.957	0.9999	0.4878	5.881	0.9994	0.2456	6.837	0.9856	–0.0132	9.153	0.4329
CS-VF-30	0.1613	8.842	8.281	0.9998	0.0289	8.842	8.881	0.9999	0.6353	4.182	0.9509	0.5315	4.362	0.9927	–0.0101	8.953	0.1900
308 K																	
CS	0.0952	7.321	7.147	0.9987	0.0173	7.321	7.849	0.9999	0.2548	5.513	0.9701	0.3371	4.547	0.9896	0.8058	0.2791	0.9967
VF	0.1317	9.321	9.034	0.9994	0.0216	9.321	9.708	0.9999	1.111	2.817	0.9994	0.3366	6.543	0.9954	–0.0029	9.356	0.0610
CS-VF-10	0.1579	7.455	7.039	0.9997	0.0341	7.455	7.533	0.9999	0.5343	3.539	0.9802	0.3946	4.146	0.9884	–0.0041	7.499	0.3546
CS-VF-20	0.2491	8.715	8.251	0.9999	0.0556	8.715	8.649	0.9999	0.6738	4.856	0.9988	0.2932	6.162	0.9165	–0.0164	8.824	0.0634
CS-VF-30	0.1135	8.543	8.053	0.9993	0.0189	8.543	8.787	0.9998	0.9031	2.519	0.9999	0.5675	3.696	0.9783	–0.0067	8.617	0.1968
318 K																	
CS	0.0678	6.821	6.542	0.9982	0.0121	6.821	7.376	0.9999	–0.0047	7.687	0.1626	–0.0029	7.356	0.0610	–0.0078	6.906	0.2298
VF	0.1116	8.821	8.303	0.9994	0.0176	8.821	9.088	0.9999	1.028	2.292	0.9882	0.8078	2.272	0.9973	–0.0078	8.906	0.2298
CS-VF-10	0.0932	6.871	6.631	0.9987	0.0184	6.871	7.286	0.9998	0.7775	1.592	0.9688	0.4747	3.033	0.9440	–0.0051	6.926	0.2964
CS-VF-20	0.1333	8.314	7.958	0.9995	0.0243	8.314	8.572	0.9999	0.9818	2.519	0.9977	0.4271	4.797	0.9927	–0.0164	8.483	0.4539
CS-VF-30	0.0952	8.267	7.685	0.9991	0.0155	8.267	8.501	0.9997	0.8020	2.291	0.9934	0.6529	2.494	0.9822	–0.0114	8.387	0.3450

The Weber and Morris internal particle diffusion model was utilized to examine the adsorption of MB dye onto the CS-VF composite microspheres. The *q*_*t*_ vs *t* ^1/2^ (Equation 10) charts indicate that internal particle diffusion is not the only determinant of the rate-limiting stage in the adsorption process. The intercept values derived from these plots, presented in Table 6 and Figure 9c, are crucial for comprehending the boundary layer impact. The intraparticle diffusion model delineates several phases in the adsorption process, each corresponding to distinct diffusion stages within the particle matrix: (i) an initial rapid external surface adsorption phase where the adsorbate transitions from the solution to the adsorbent surface; (ii) a subsequent phase characterized by the gradual diffusion of the solvent into the adsorbent’s pores; and (iii) a concluding phase marked by slow diffusion from larger pores to smaller pores.^88,89^ Figure 9c elucidates these phases, demonstrating the intricate nature of the adsorption mechanism. During the initial phase, the adsorption capacity increased by *t* ^1/2^, which was ascribed to the fast migration of dye molecules toward the surfactants and boundary layer diffusion. Electrostatic attraction and hydrogen bonding interactions between amino/hydroxyl groups and dye molecules predominated this phase, resulting in increased adsorption rates. In the subsequent phase, the adsorption rate diminished owing to the escalating intraparticle diffusion resistance as dye molecules progressively infiltrated the internal pores. In the last phase, the adsorption rate progressively diminished due to the saturation of active sites and the reduction of diffusion repulsive forces until equilibrium was attained. The divergence of the model curves from the origin validated the synergistic regulation of liquid film diffusion and intraparticle diffusion throughout the adsorption process.^90^

The kinetics of Boyd were examined to elucidate the influence of particle and film diffusion on the comprehensive removal of MB dye by composite microspheres. In Figure 9d, the linear graph does not cross the origin. This signifies the presence of additional rate-limiting stages beyond the intraparticle diffusion in the adsorption process. The use of adsorption kinetic data to the Boyd kinetic model facilitated additional confirmation of the rate-limiting stage in the real adsorption process depicted in Figure 9d. The linear configuration of the graphs in Figure 9d, along with their nonintersection with the origin, indicates that film diffusion regulates the adsorption process.^91^

On the other hand, the feasibility of the kinetic models was also evaluated using the error functions listed in Table 2, and the results were listed in Table 7. As seen in Table 7, the analysis of the error functions indicated that the *pseudo-second-order* kinetic model was the most suitable kinetic model for MB adsorption onto adsorbents. This is also evident from the closeness and high correlation values between *q*_e,cal_ and *q*_e,exp_. Furthermore, an examination of the error functions given in Table 7 reveals that the intraparticle diffusion kinetic model is the most suitable kinetic model after the *pseudo-second-order* kinetic model.

**Table 7:** Error Functions for the Adsorption of MB Dye onto CS, VF, CS-VF-10, CS-VF-20, and CS-VF-30 Composite Microbeads at Different Temperatures

	nonlinear *pseudo-first order*				nonlinear *pseudo-second order*				intraparticle diffusion			
temperature	SSE	ARE	SD	*χ* ^2^	SSE	ARE	SD	*χ* ^2^	SSE	ARE	SD	*χ* ^2^
298 K												
CS	1.573	5.824	0.4181	0.2692	0.2654	2.209	0.1717	0.0467	1.313	2.258	0.3819	0.1977
VF	2.174	5.154	0.4914	0.2636	0.3901	2.047	0.2082	0.0499	1.301	4.107	0.3802	0.1502
CS-VF-10	1.894	6.292	0.4588	0.2744	0.5836	3.489	0.2547	0.0874	0.3668	2.408	0.2019	0.0507
CS-VF-20	1.878	5.108	0.4568	0.2301	0.4295	2.457	0.2185	0.0546	0.5316	2.651	0.2431	0.0641
CS-VF-30	3.977	8.501	0.6647	0.5847	1.187	4.498	0.3632	0.1741	1.254	3.992	0.3733	0.1606
308 K												
CS	0.7649	4.655	0.2915	0.2059	0.1501	2.255	0.1291	0.0294	3.768	10.48	0.6471	0.6666
VF	1.961	5.587	0.4667	0.3372	0.2865	2.041	0.1784	0.0467	3.834	7.641	0.6527	0.4978
CS-VF-10	2.626	8.286	0.5402	0.4889	0.7292	4.361	0.2846	0.1362	1.042	4.729	0.3402	0.1588
CS-VF-20	1.679	5.385	0.4319	0.2192	0.3488	2.363	0.1969	0.0453	0.8511	3.576	0.3075	0.1101
CS-VF-30	3.379	8.009	0.6127	0.6128	0.8597	3.989	0.3091	0.1518	2.001	6.103	0.4716	0.2849
318 K												
CS	2.189	9.268	0.4931	0.5655	0.7959	4.969	0.2974	0.1656	2.394	8.819	0.5158	0.4717
VF	4.171	8.689	0.6808	0.6774	1.299	4.553	0.3799	0.1987	2.434	6.048	0.5201	0.3368
CS-VF-10	1.589	6.847	0.4202	0.4638	0.4529	4.091	0.2243	0.1186	2.394	8.661	0.5158	0.4332
CS-VF-20	2.033	6.635	0.4753	0.3644	0.3461	2.754	0.1961	0.0599	2.439	6.993	0.5206	0.3548
CS-VF-30	4.545	9.971	0.7106	0.9176	1.585	5.888	0.4197	0.3064	1.543	5.374	0.4141	0.2218

### 3.7 Adsorption Mechanism

Figure 10 illustrates the suggested MB dye adsorption method on CS-VF-30 composite microbeads. The suggested adsorption mechanism for MB elimination is based on a collection of corroborative studies demonstrating the simultaneous functioning of several interactions. The pH_pzc_ of 7.08 and the observation of greatest retention at pH ≥ 7 indicate that the surface possesses a net negative charge, resulting in a predominant electrostatic attraction to cationic MB. This interpretation aligns with the reported reduction in capacity due to increasing ionic strength in salinity experiments and corroborates the electrostatic role. The observed shifts and reductions in intensity of the −OH (3285→3289 cm^–1^), C=O/N–H (1732, 1634→1645/1558 cm^–1^), −CH_3_/–COO^–^ (1393→1375 cm^–1^), S=O (1131→1150 cm^–1^), and C–O (1023→1027 cm^–1^) bands in FT-IR indicate interactions between hydroxyl, carboxyl, amine, and sulfate groups with MB. Notably, the shift from 1393→1375 cm^–1^ to a lower frequency implies torsional relaxation, which is consistent with an increase in local hydrogen bonding and electron density following complexation. These modifications facilitate hydrogen bonding (including −OH/–NH···N/–S functionalities) and electrostatic interactions, together with potential π–π stacking between the aromatic rings of MB and the aromatic sites within the composite. The concurrent filling and smoothing of surface pores postadsorption in SEM, along with the identification of Cl from MB in EDS mapping, unequivocally validate the retention of the dye on the surface. Consequently, the adsorption of MB on CS-VF-30 is suggested to occur through a multifaceted interaction mechanism, as corroborated by FT-IR shifts, salinity sensitivity, and EDS-SEM results, which include (i) pH-dependent electrostatic attraction, (ii) hydrogen bonding and coordinative/ionic interactions involving −OH/–COO^–^/–NH/–SO_3_ groups, and (iii) a synergistic effect of π–π stacking among aromatic rings.

**Figure 10:**
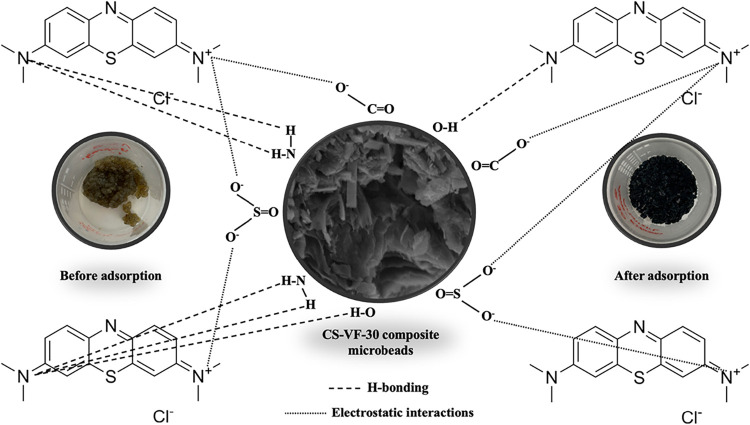
Illustration of the possible interaction between the CS-VF-30 surface and MB including electrostatic interactions and hydrogen bonding.

### 3.8 Adsorption Thermodynamics

We estimated the adsorption thermodynamic parameters, such as the change in free energy (Δ*G* ^o^), change in enthalpy (Δ*H* ^o^), and change in entropy (Δ*S* ^o^), to explain the temperature-dependent variation of MB adsorption onto VF and CF-VF composites. As seen in Figure 11a below, the values of Δ*H* ^o^ and Δ*S* ^o^ were calculated from the slope and intercept of the plots of ln *K*e^o^ and 1/*T*, respectively. Table 8 displays the results of the calculations for thermodynamic parameters, including free energy (Δ*G* ^o^), enthalpy (Δ*H* ^o^), and entropy (Δ*S* ^o^). In support of the findings in the adsorption isotherms, the negative values of Δ*G* ^o^ and Δ*H* ^o^ verify that the adsorption process is exothermic, spontaneous, and practicable. Weak linkages between the adsorbent surface and the adsorbate are formed, as indicated by the study’s lower entropy and enthalpy change values.^48,92^ However, according to Arrhenius theory, a linear graph was created using eq 14 and *pseudo-second-order* rate constants (*k*_2_) between the values of 1/T and ln *k*_2_, as seen in Figure 11b. The adsorption process can be considered exothermic based on the activation energy (*E*_a_) values that are determined from the graph’s slope and provided in Table 8.

**Figure 11:**
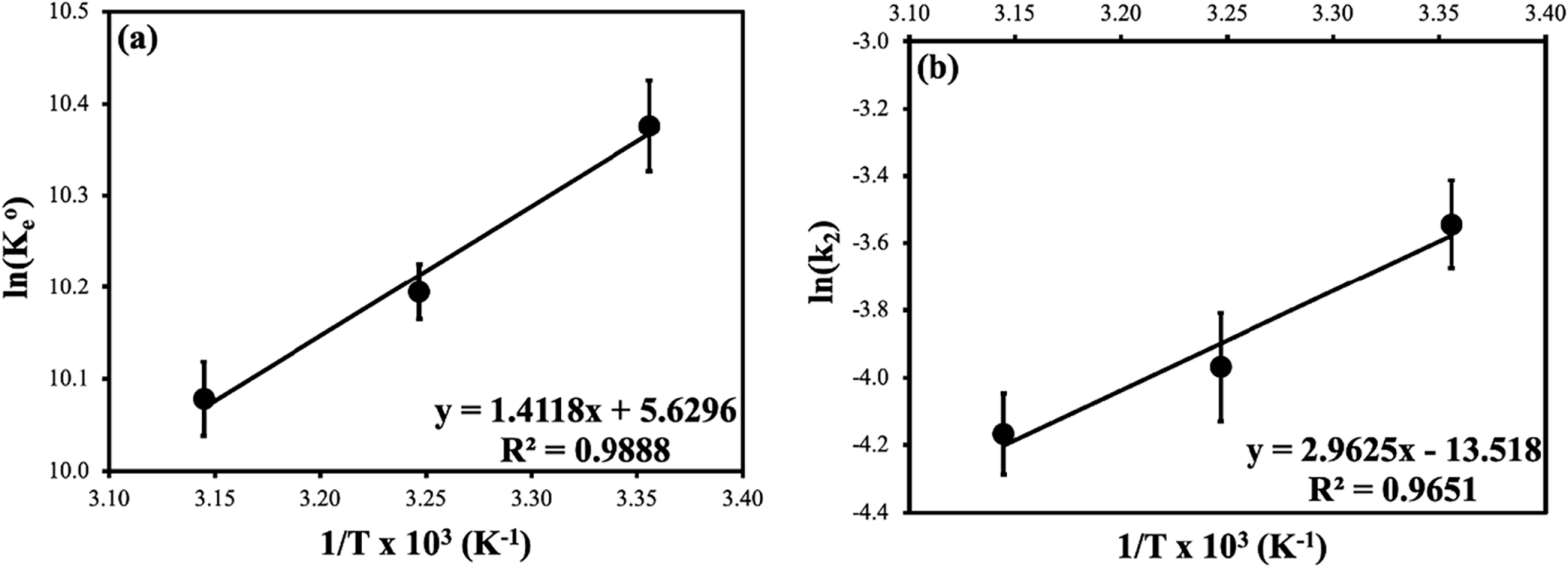
Linear Van’t Hoff plot (a) and Arrhenius plot (b) of MB dye adsorption onto CS-VF-30 composite microbeads. Error bars indicate standard deviation from triplicate measurements (*n* = 3).

**Table 8:** Thermodynamic Parameters of MB Dye onto CS, VF, CS-VF-10, CS-VF-20, and CS-VF-30 Composite Microbeads

				Δ*G*° (kJ/mol)		
thermodynamic parameters	Δ*H*° (kJ/mol)	Δ*S*° (J/mol/K)	*E*_a_ (kJ/mol)	298 K	308 K	318 K
CS	–3.128	75.89	–49.71	–25.74	–26.51	–27.26
VF	–45.46	–49.80	–40.09	–30.62	–30.12	–29.62
CS-VF-10	–6.781	62.15	–52.20	–25.30	–25.92	–26.54
CS-VF-20	–15.12	39.64	–38.09	–26.93	–27.33	–27.73
CS-VF-30	–11.74	46.80	–24.63	–25.69	–26.15	–26.62

### 3.9 Reusability

The reusability of the CS-VF-30 composite microbeads for MB adsorption is demonstrated in Figure 12. Under comparable circumstances, the CS-VF-30 composite microbeads were recycled six times. After removing the adsorbent by decantation without centrifugation, distilled water and ethanol were used for washing. Following the sixth cycle, the adsorption percentage dropped from 85.12 to 63.45%, as shown in Figure 12. This slight reduction suggested that the produced composite microbeads could be utilized repeatedly. The presence of some heterogeneities on the surface of CS-VF-30 composite microspheres is indicated by the reduced desorption efficiency in comparison to the adsorption efficiency in the adsorption/desorption cycles. Additionally, these regions exhibit binding energies that are greater than the typical range. Because of this circumstance, it is possible that dye molecules will bind to these regions, thereby forming strong bonds and making the desorption process more challenging or even impossible.^93^

**Figure 12:**
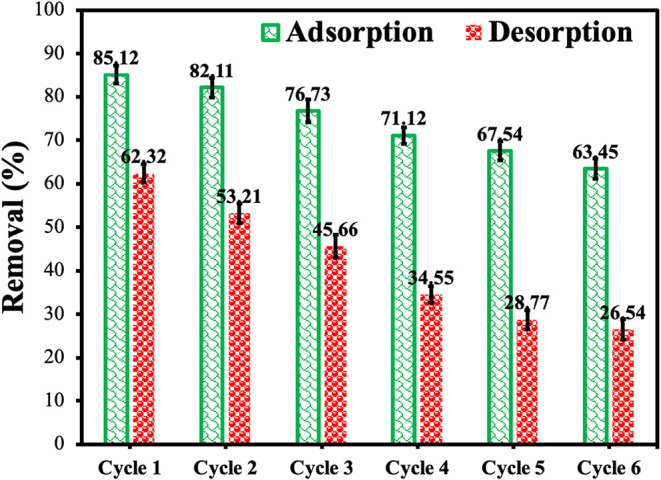
Adsorption–desorption cycles of CS-VF-30 composite microbeads for MB dye removal. Error bars indicate standard deviation from triplicate measurements (*n* = 3).

Figure 13 illustrates the XRD pattern before and after reusability (Figure 13a), SEM image after reusability (Figure 13b), and FT-IR spectra after reusability (Figure 13c). In Figure 13a, the diffraction peaks detected at roughly 2θ = 9–10° and 2θ ≈ 20° are indicative of chitosan and other cellulose-based lignocellulosic composites, hence affirming the semicrystalline structure of the CS–VF–30 microbeads. The peak at 9–10° corresponds to the hydrated crystalline form of chitosan, resulting from the orderly organization of polymer chains via intramolecular hydrogen bonding. The prominent peak between 19 and 21° is commonly recognized as the primary reflection of semicrystalline chitosan and cellulose I structures. These positions align with previously described XRD patterns of chitosan films, chitosan-based composites, and lignocellulosic materials.^85,94,95^ In the study conducted by Bellaj et al., the peaks observed at 20.07° at 2θ (°) diffraction angles in the XRD pattern of pure chitosan confirmed the semicrystalline polymer.^1,74^ On the other hand, when the XRD spectrum was examined after reusability, it was seen that the structure was not damaged. In a similar manner, Kurczewska demonstrated that pure CS beads exhibited two broad reflections at 10.4 and 20.3°. These reflections were due to hydrogen bonding between the amino and hydroxyl groups. Kurczewska accomplished this by working with chitosan.^96^ When the postreusability SEM image in Figure 13b is examined, it is observed that the surface is similar to the postadsorption SEM images shown in Figure 3b, and the structure is preserved even after the reusability process. When the FT-IR spectrum after reusability in Figure 13c is examined, it is observed that the structure does not change after the reusability process and is almost identical to the spectrum after adsorption shown in Figure 2. When the FT-IR spectrum is examined, it is observed that the peaks show small shifts. These shifts indicate that the adsorption process also occurs effectively during the reusability stages.

**Figure 13:**
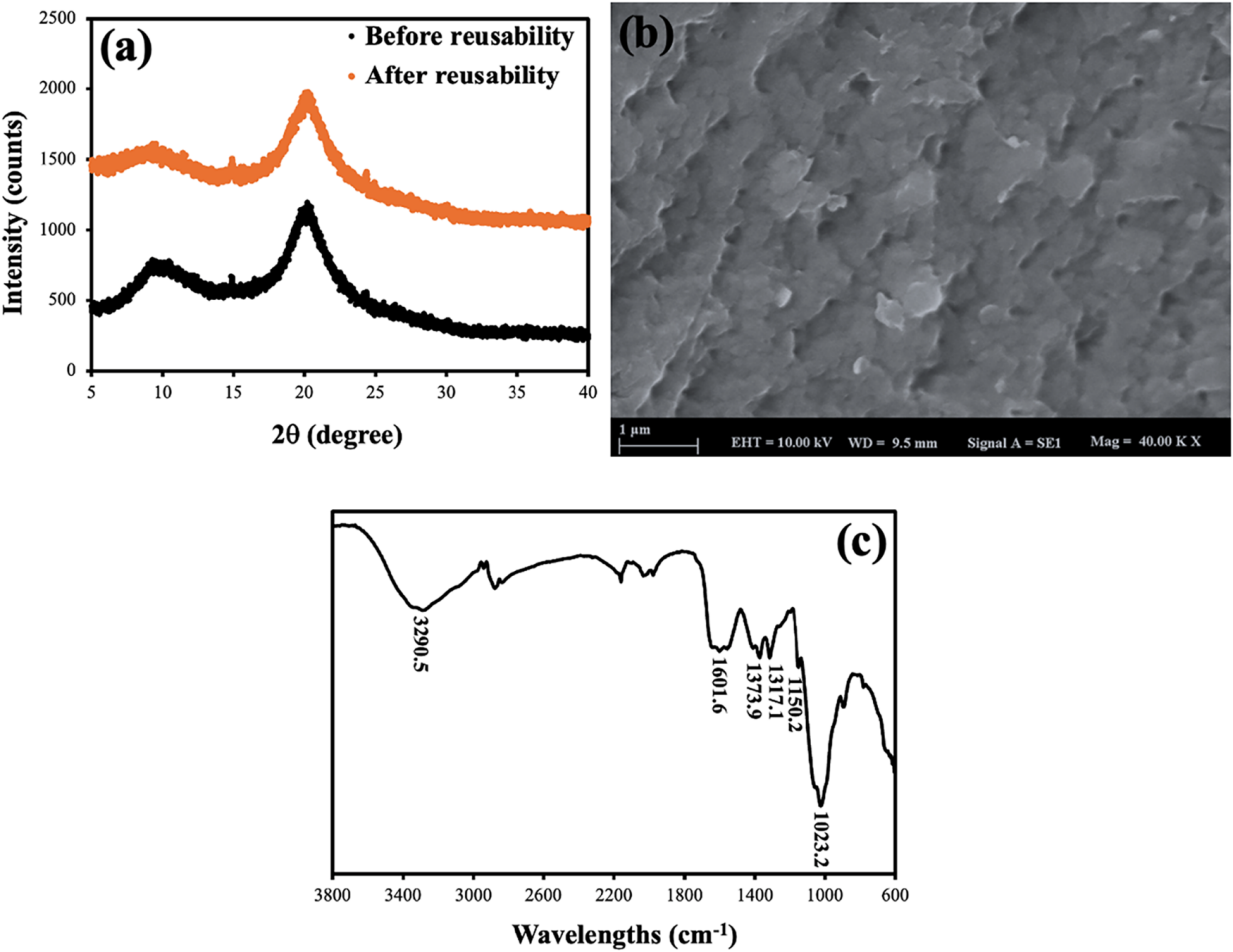
XRD patterns (detector: PIXcel3D, anode material: Cu, intended wavelength type: K-α1 radiation: I = 1.54° Å, generator settings: 40 mA and 45 kV, step size [°2θ]: 0.0130, start position [°2θ]:10.0012, end position [°2θ]: 29.9832) of CS-VF-30 composite microbeads before and after reusability (a), SEM images (b), and FT-IR spectra (c) after reusability.

### 3.10 Real Wastewater Experiments

This work examined the adsorption efficacy of MB dye on CS-VF-30 composite microspheres utilizing diverse water samples in a controlled laboratory environment. The outcomes are illustrated in Figure 14. To eliminate the MB dye, an adsorbent comprising 0.010 g of CS-VF-30 composite microspheres in 50 mL of aqueous solution was agitated at 120 rpm for 90 min. Figure 14 unequivocally demonstrates that distilled water has the maximum adsorption effectiveness for the MB dye. The presence of diverse ions in tap and drinking water adversely impacted the adsorption process of MB dye. The adsorption efficiency for MB dye in tap water diminished from 88.4 to 87.6%, while in drinking water, it declined from 88.4 to 81.2%. Fluctuating ionic charges in the aqueous environment might adversely impact the adsorption of MB dye. The results indicate that CS-VF-30 composite microspheres demonstrate considerable efficacy in diverse water samples.

**Figure 14:**
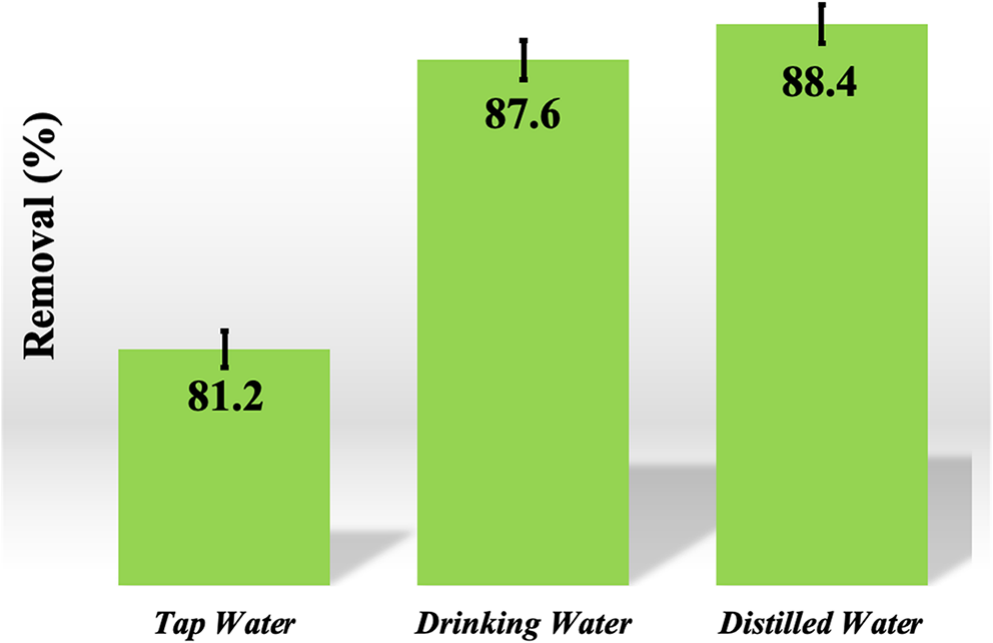
Removal performance of MB dye onto CS-VF-30 composite microbeads in different water samples. Error bars indicate standard deviation from triplicate measurements (*n* = 3).

## 4 Conclusions

This work effectively developed new CS-VF composite microbeads by utilizing straightforward procedures. Transforming biomass waste into usable adsorbents in a zero-waste approach effectively addresses disposal issues and enhances the valorization of natural resources. The adsorption of MB onto CS-VF-30 composite microbeads was accomplished by optimizing all critical elements influencing the adsorption process, including the pH, dosage, contact duration, initial concentration, and temperature. The peak removal efficiency of MB on the adsorbent was 85.12% at pH 7, a dosage of 0.1 g, a contact duration of 90 min, a stirring speed of 120 rpm, and a temperature of 298 K. The composite microbeads were characterized by FT-IR, SEM, XRD, and pH_pzc_ studies. FT-IR research indicated that diverse functional groups on the composite microbeads’ surface were capable of adsorption. Four nonlinear adsorption isotherm models were assessed: Langmuir, Freundlich, D-R, and Temkin. The Langmuir isotherm model demonstrated the optimal fit based on correlation coefficients and error functions, indicating homogeneous monolayer adsorption with a maximum capacity of 45.79 mg/g. The kinetics of adsorption were examined by utilizing *pseudo-first-order* and *pseudo-second-order* models. The pseudo-second-order kinetic model exhibited the optimal fit. The negative values of Δ*H* ^o^ and Δ*G* ^o^ revealed that the adsorption process was both exothermic and spontaneous. The adsorption process was efficiently facilitated by electrostatic, hydrogen bonding, and π–π interactions. Moreover, reusability trials demonstrated that the composite microbeads remained effective until the conclusion of the sixth cycle. This research underscores the efficacy of composite microbeads derived from waste *Vitis vinifera* L. biomass and chitosan biopolymer as proficient adsorbents for cationic dyes.

## Data Availability

Data used is available throughout the manuscript text.
